# Serum levels of the proinflammatory cytokine interleukin-6 vary based on diagnoses in individuals with lumbar intervertebral disc diseases

**DOI:** 10.1186/s13075-015-0887-8

**Published:** 2016-01-07

**Authors:** Kathryn T. Weber, D. Olivier Alipui, Cristina P. Sison, Ona Bloom, Shaheda Quraishi, M. Chris Overby, Mitchell Levine, Nadeen O. Chahine

**Affiliations:** The Feinstein Institute for Medical Research, North Shore-LIJ Health System, Manhasset, NY USA; Department of Molecular Medicine, Hofstra North Shore-LIJ School of Medicine, Hempstead, NY USA; Department of Physical Medicine and Rehabilitation, Hofstra North Shore-LIJ School of Medicine, Hempstead, NY USA; Department of Neurosurgery, Hofstra North Shore-LIJ School of Medicine, Hempstead, NY USA; Department of Orthopedic Surgery, Hofstra North Shore-LIJ School of Medicine, Hempstead, NY USA; Department of Population Health, Hofstra North Shore-LIJ School of Medicine, Hempstead, NY USA

**Keywords:** Intervertebral disc, Back pain, Inflammatory cytokines, Matrix metalloproteinase, Biomarkers, Disc herniation, Spinal stenosis, Degenerative disc disease, Clinical research, Epidural steroid injections

## Abstract

**Background:**

Many intervertebral disc diseases cause low back pain (LBP). Proinflammatory cytokines and matrix metalloproteinases (MMPs) participate in disc pathology. In this study, we examined levels of serum cytokines and MMPs in human subjects with diagnoses of disc herniation (DH), spinal stenosis (SS), or degenerative disc disease (DDD) relative to levels in control subjects. Comparison between subjects with DH and those with other diagnoses (Other Dx, grouped from SS and DDD) was performed to elaborate a pathological mechanism based on circulating cytokine levels.

**Methods:**

Study participants were recruited from a spine neurosurgery practice (*n* = 80), a back pain management practice (*n* = 27), or a control cohort (*n* = 26). Serum samples were collected before treatment and were assayed by multiplex assays for levels of interleukin (IL)-1β, IL-2, IL-4, IL-6, IL-8, IL-10, IL-12p70, IL-13, interferon-γ, tumor necrosis factor-α, MMP-1, MMP-3, and MMP-9. Inflammatory and degradative mediator levels were compared for subjects with LBP and control subjects, by diagnosis and by treatment groups, controlling for effects of sex, age, and reported history of osteoarthritis. Spearman’s correlation coefficient was used to examine relationships with age, body mass index (BMI), symptom duration, and smoking history.

**Results:**

Serum levels of IL-6 were significantly higher in subjects with LBP compared with control subjects. Participants with LBP due to Other Dx had significantly higher levels of IL-6 than DH and controls. Serum levels of MMP-1 were significantly lower in LBP subjects, specifically those with DH, than in control subjects. Positive correlations were found between IL-6 levels and BMI, symptom duration, and age. MMP-1 levels were positively correlated with age.

**Conclusions:**

The findings of the present clinical study are the results of the first examination of circulating cytokine levels in DDD and SS and provide evidence for a more extensive role of IL-6 in disc diseases, where patients with DDD or SS have higher serum cytokine levels than those with DH or control subjects. These findings suggest that LBP subjects have low-grade systemic inflammation, and biochemical profiling of circulating cytokines may assist in refining personalized diagnoses of disc diseases.

## Background

Back pain causes a significant burden on both the healthcare system and the economy. As the second most common cause of physician visits in the United States, low back pain (LBP) contributes an estimated $50 billion to 100 billion in direct healthcare spending [1–4]. Affecting an estimated 40–80 % of people at any point during their lifetime, LBP is a leading cause of disability worldwide [5]. Clinically, LBP is a complicated amalgam of multiple diseases with an unpredictable response to treatment. LBP is caused by multiple triggers with similar clinical presentation, and physical examination is rarely diagnostic. Some of the most common diagnoses for LBP include intervertebral disc herniation (DH), spinal stenosis (SS), and degenerative disc disease (DDD).

The intervertebral disc (IVD) is the load-bearing tissue between the vertebral bodies of the spine. The disc is composed of a gelatinous nucleus pulposus (NP) in the center surrounded by a fibrous annulus fibrosis (AF) tissue in the periphery. DH is the focal displacement of disc material beyond the limits of the IVD disc space [6]. DH can irritate nearby nerves and result in pain, numbness, or weakness in the legs. SS is a pathologic narrowing of the spinal canal due to hypertrophy of the ligamentum flavum and is associated with paresthesia, pain, and symptoms of neurologic compromise [7, 8]. DDD includes disc pathology such as desiccation and loss of disc height, diffuse disc bulging, fissures, osteophytes, or endplate sclerosis without meeting a specific indication for herniation or stenosis [6]. Currently, the best prognostic indicators for LBP are based on the underlying diagnosis. In some cases, DH will remodel spontaneously with nonoperative treatment and patients will experience resolution of painful symptoms [9]. In contrast, age is a primary risk factor for SS [10], so people who develop it at younger ages have longer durations of symptoms and worse prognoses [11].

Disruption of the extracellular matrix (ECM) of the disc, such as loss of proteoglycans (PGs) in the NP, increase in degenerative fibrillation in NP and AF, and loss of water content in the NP, are hallmarks of IVD degeneration [12–16]. This has previously been shown to be associated with upregulation of matrix metalloproteinases (MMPs) such as MMP-1, MMP-3, MMP-7, and MMP-13 expression and activity, whose levels correlate with increasing degenerative severity [17–20]. Other catabolic proteases, such as a disintegrin and metalloprotease with thrombospondin motifs (known as ADAMTS), high temperature requirement serine protease A1, and cathepsins, also promote aggrecan turnover and degenerative changes in the disc [21–26]. The increased catabolism of aggrecan is a principal pathological process that leads to degeneration [27], compromising functional integrity of the IVD. Several etiological factors serve as primary initiating events that lead to the abnormal production of catabolic molecules by IVD cells [28]. These factors include aging, genetic predisposition, smoking, infection, abnormal biomechanical loading, and inadequate nutrient supply [28–35]. Inflammatory cytokines play a major part in the pathogenesis of IVD degeneration by promoting ECM breakdown. Disc-derived cells have the capacity to secrete proinflammatory cytokines in the absence of inflammatory cells, supporting the hypothesis that disc-derived cells are capable of initiating or amplifying an inflammatory process. Degradation has been associated with increased expression of inflammatory mediators such as nitric oxide and prostaglandin E_2_ by disc cells [36, 37]. Many proinflammatory cytokine levels are also elevated in degenerated or herniated IVDs [38–41], including tumor necrosis factor-α (TNF-α), interleukin (IL)-1, IL-6, IL-17, and interferon-γ (IFN-γ) [39, 42, 43]. Inflammatory cytokines also promote recruitment of immune cells to the disc in disease processes. Infiltration and activation of immune cells result in amplification of an inflammatory response and the release of neurotrophic factors that contribute to neoinnervation of the disc or nerve sensitization of spinal nerves [28, 44–46].

Levels of proinflammatory cytokines implicated in disc remodeling, nerve sensitization, and local pathology in the spine have been studied systemically in participants with LBP; however, the findings are inconsistent. Bisby et al. did not find major differences in serum cytokine levels of patients with DH and sciatica compared with normal levels that were reference values used by a local clinical immunological laboratory in routine clinical analyses [47]. Pedersen et al., however, observed higher serum levels of IL-6 and IL-8 in participants with DH who reported higher pain scores than participants with lower pain scores [48]. Moreover, Schistad et al. observed that elevated serum IL-6 levels at presentation were associated with worse outcomes in recovery from DH at 1-year follow-up [49]. While DH has some relationship with circulating levels of proinflammatory cytokines, changes in mediator profiles with increasing disease severity and in diagnoses other than DH are unknown. Aging, higher body mass index (BMI), and smoking history are all risk factors for LBP [5] and are associated with poor prognosis [50]. Aging is associated with increased levels of circulating cytokines and proinflammatory markers. Increased levels of IL-6, IL-1, or TNF-α are associated in older patients with frailty, sarcopenia, cardiovascular events, and neurodegenerative diseases, which increase the risk of morbidity and mortality [51]. Obesity is also associated with increased serum levels of IL-6 and TNF-α, among other cytokines and chemokines [52]. Tobacco use generates a systemic proinflammatory state that is characterized by serum elevations of TNF-α and IL-1, among other cytokines [53].

The goal of this study was to examine serum levels of proinflammatory cytokines and MMPs in participants with LBP due to DH, SS, or DDD relative to control participants. We hypothesized that the proinflammatory mediators known to participate in IVD pathology would be elevated systemically relative to control participants. We further hypothesized that mediator levels would differ between diagnoses and change with the natural history of disease. Because LBP and proinflammatory cytokines change with age, BMI, tobacco use, duration or intensity of pain symptoms, and disability, we analyzed the potential relationships between these clinical and demographic factors and circulating inflammatory mediator levels in DH, SS, and DDD.

## Methods

### Participants

This study was approved by the institutional review board of North Shore-LIJ Health System. One hundred seven participants were recruited from two clinical practices (back pain management, spinal neurosurgery). Written informed consent was obtained from all participants before enrollment. Similar inclusion criteria were used for identification of participants in each cohort. For the back pain management cohort, participants who were 18 years of age or older and required interlaminar epidural steroid injection (ESI) in the lumbar spine region (L1-L2 to L5-S1) for SS, DH, or DDD were recruited (ESI cohort; *n* = 27). Participants were excluded if they had a history of previous lumbar surgery, epidural corticosteroid injection within the last 6 months, known inflammatory condition (e.g., rheumatoid arthritis [RA], systemic lupus erythematosus, gout, osteomyelitis, other infections), or a history of cancer or were pregnant or breastfeeding at the time of recruitment. For the surgical cohort, participants who were 18 years of age or older, required surgery in the lumbar spine (L1-L2 to L5-S1), including lumbar fusion, discectomy, laminectomy, and/or minimally invasive discectomy, were recruited (surgery cohort; *n* = 80). Participants requiring revision surgery in the lumbar spine who had a history of prior discography procedure at the same spinal level or known inflammatory conditions or a history of cancer were excluded. For all subjects, other joint health status (e.g., osteoarthritis [OA] of the knee or other joints) was not surveyed or used as an exclusion criterion. On the basis of medical records, 18 of 107 study participants had a reported current or past medical history of arthritis, OA, or related joint pain. The effect of OA history was controlled for in the data analysis (see Statistical analysis section below).

Before clinical intervention, blood samples were obtained at the time of the procedure. In surgical participants, blood samples were collected before administration of anesthesia. One 10-ml sample of whole blood was collected (BD Vacutainer 367820; BD, Franklin Lakes, NJ, USA) and maintained on ice until processing. For each participant, serum was isolated by centrifugation and aliquots were stored at −80 °C until biochemical analysis. Clinical data, including basic demographic information, underlying diagnosis, duration of symptoms, and treatment history (particularly prior history of ESI), were collected from the participants and their medical records. Magnetic resonance imaging (MRI) scans of T2-weighted spin-echo images at the spinal level of the procedure were evaluated for degeneration severity. Severity was classified using Pfirrmann grade (1–5) [54]. Grade 1 represents a healthy IVD exhibiting a bright hyperintense white signal intensity and normal disc height, and grade 5 represents a hypointense black signal intensity with no clear distinction between the NP and AF and a collapsed disc space. Owing to lack of access to imaging scans for a small number of participants (*n* = 4), radiology reports were used to assign Pfirrmann grades.

Serum levels of biochemical factors were also measured in age- and gender-matched control participants (control cohort; *n* = 26). Written informed consent was obtained from control participants before recruitment. Participants 18 years of age or older with no history of spinal injury or inflammatory conditions were included in the control cohort. Venipuncture was performed to collect serum using the same methods described for participants with LBP. Age and gender information was collected for each participant.

### Biochemical analysis

Serum levels of cytokines and MMPs were measured using commercially available multiplex electrochemiluminescence immunoassays (Meso Scale Discovery [MSD], Rockville, MD, USA). The assay was performed according to the manufacturer’s recommendations. Samples were run in duplicate to measure IL-1β, IL-2, IL-4, IL-6, IL-8, IL-10, IL-12p70, IL-13, IFN-γ, TNF-α, MMP-1, MMP-3, and MMP-9. For the cytokine multiplex (V-PLEX Proinflammatory Panel 1; MSD), samples were diluted to 1:2 in Diluent 2 (MSD) and 50 μl were added and incubated at 4 °C overnight. For the MMPs, a multiplex plate (Human MMP 3-Plex Ultra-Sensitive kit; MSD) was blocked for 30 minutes before use with 25 μl of Diluent 2. Samples were diluted 1:10 in Diluent 2, and 25 μl of diluted sample were added to the MMP plate and allowed to incubate for 2 h at room temperature. Following the initial incubation, plates were washed with 0.05 % Tween in phosphate-buffered saline, and 25 μl of detector antibody were added to each well. Plates were incubated for 2 h at room temperature, then washed again, and read with 2× read buffer using MSD SECTOR Imager 2400 plate reader. The lower and upper limits of detection (LLOD and ULOD, respectively) were computed for each assay, and the percentage of samples with concentrations at or above the LLOD (% detected) are reported (Table 1). For statistical analysis, values that were below the LLOD were assigned a value that was half of the LLOD, as previously described [55].

**Table 1:** Lower and upper limits of detection (LLOD and ULOD, respectively) for measured biochemical factors in serum samples (pg/ml)

Biochemical factor	LLOD (pg/ml)	ULOD (pg/ml)	% Detected
IL-1β	0.069	495	9%
IL-2	0.055	1460	59%
IL-4	0.025	198	28%
IL-6	0.068	767	98%
IL-8	0.073	498	100%
IL-10	0.058	334	99%
IL-12p70	0.078	1460	42%
IL-13	0.50	466	41%
TNF-α	0.085	312	100%
IFN-γ	0.30	1460	98%
MMP-1	4.44	100,000	100%
MMP-3	5.10	100,000	100%
MMP-9	37.73	500,000	100%

Percentage detected (% detected) is the percentage of samples with concentrations at or above the LLOD

### Statistical analysis

The mean and standard deviation of continuous demographic or clinical variables (age, BMI, symptom duration, smoking history, and OA history) are reported. The gender distribution in the control and LBP cohorts was compared using Fisher’s exact test, and subject age was compared using the Mann-Whitney *U* test. All analyses of biochemical factors were performed on the logarithmic transformation of analyte levels to conform to standard model assumptions. Results were then back-transformed and presented using the geometric means with their corresponding standard errors. Levels of cytokines and MMPs were compared between control and LBP subjects using analysis of covariance (ANCOVA), controlling for age and gender. The interaction effect of gender and subject cohort (control or LBP) was assessed in each model. Analysis of cytokines and MMPs between the control, ESI, and surgery groups was performed using ANCOVA, controlling for age and gender, and specific comparisons were made using Tukey’s honest significant difference (HSD) post hoc test. Differences between diagnostic groups were examined using ANCOVA, controlling for age, gender, and OA history. The diagnosis groups were either participants with DH or with other diagnoses (Other Dx) pooled from participants with SS or DDD. The interaction effect of gender and diagnosis group was assessed in each model. Specific comparisons of biochemical factors between the control, DH, and Other Dx groups was performed using Tukey’s HSD post hoc test. Age, BMI, smoking history (in pack-years), and symptom duration were compared between diagnosis groups using the Mann-Whitney *U* test. Gender distribution and OA history of subjects by diagnosis was compared using Fisher’s exact test. Spearman’s correlation coefficients were calculated to determine the relationship between analyte levels and age, BMI, duration of pain symptoms, or smoking history (pack-years), and the resulting Spearman’s coefficients (ρ) and *p* values are reported. Analyses were performed using SAS version 9.3 (SAS Institute, Cary, NC, USA) and Statistica (StatSoft, Tulsa, OK, USA) software. Significance testing was performed with *p* < 0.05.

## Results

### Participant characteristics

In this study, we recruited participants (*N* = 133) with an average age of 50 ± 14 years and gender distribution of 43 % male (*n* = 57) and 57 % female (*n* = 76) (Table 2). A comparison of the age and gender demographics of the control and LBP cohorts is presented in Table 2. The control cohort was 39 % female and 61 % male, and the LBP cohort was 62 % female and 38 % male (*p* = 0.046), though both cohorts had comparable age distributions. The demographics and comparison of the treatment cohorts (ESI vs. surgery) are presented in Table 3. Participants in the ESI cohort were 55 ± 18 years old; 63 % were female and 37 % were male. Participants in the surgery cohort were 49 ± 13 years old; 61 % were female and 39 % were male. In a comparison between the control, ESI, and surgery cohorts, no significant differences in age (*p* = 0.14) or gender (*p* = 0.12) distribution were observed. The ESI cohort had a BMI of 29.8 ± 6.3, reported symptom duration of 20 ± 23 months and had an average smoking history of 5 pack-years. Participants in the surgery cohort had a BMI of 28.9 ± 5.6, reported symptom duration of 24 ± 42 months, and had an average smoking history of 7.6 pack-years (Table 3). No signficant differences were found in BMI, symptom duration, or smoking history between the ESI and surgery cohorts (*p* > 0.5). A significant difference was observed, however, in the breakdown of diagnoses in the ESI and surgery cohorts (*p* < 0.05). A significantly greater number of participants in the surgery cohort were diagnosed with DH (74 %) than in the ESI cohort (48 %) (Table 3). The distribution of Pfirmann grades in the surgery and ESI cohorts is presented in Table 3.

**Table 2:** Summary of participant demographics in control and LBP cohorts

	Control	LBP	p-value
	N=26	N=107	
Gender distribution			
Male	16 (61%)	41 (38%)	0.046*
Female	10 (39%)	66 (62%)	
Age (mean ± SD)	48 ± 12	51 ± 15	0.33

Statistical comparison of control vs. LBP cohorts was performed, and p-values are presented

*p<0.05

**Table 3:** Summary of participant demographics in ESI and surgery treatment cohorts

	ESI	Surgery	p-value
	N=27	N=80	
Gender distribution			
Male	10 (37%)	31 (39%)	0.89
Female	17 (63%)	49 (61%)	
Age (mean ± SD)	55 ± 18	49 ± 13	0.07
BMI (mean ± SD)	29.8 ± 6.3	28.9 ± 5.6	0.53
Diagnosis breakdown (%)			
DH	13 (48%)	59 (74%)	0.046*
Other Dx	14 (52%)	21 (26%)	
Spinal Stenosis	7	19	
DDD	7	2	
Symptom Duration (months, mean ± SD)	20 ± 23	24 ± 42	0.71
Smoking History (pk yrs, mean ± SD)	5.0 ± 8.9	7.6 ± 13.2	0.51
Pfirmann Grade Distribution (Number of subjects per grade)			
Grade I	1	0	
Grade II	2	4	0.006*
Grade III	3	12	
Grade IV	16	38	
Grade V	5	26	

Statistical comparison of ESI vs. surgery cohorts is presented (p-value)

*p<0.05

### Biochemical factors in LBP and control cohorts

**Fig. 1:**
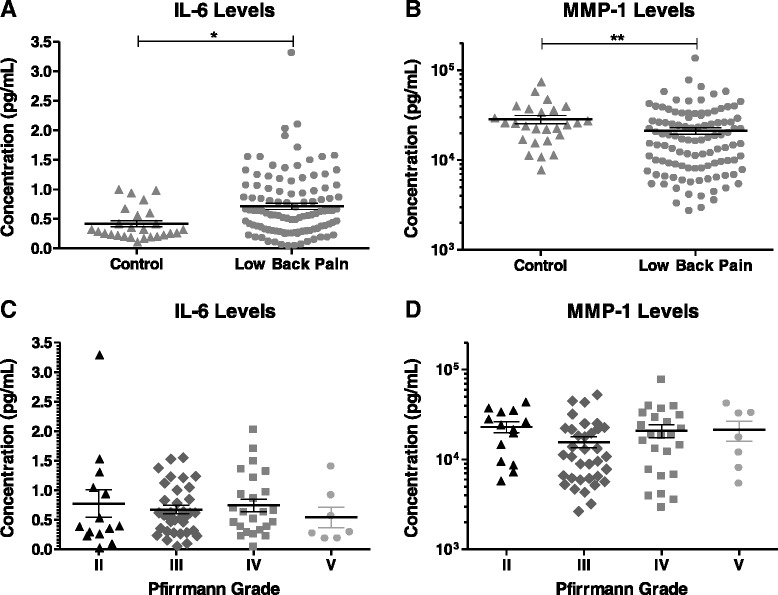
Comparison of serum levels of (**a**) interleukin (IL)-6 and (**b**) matrix metalloproteinase (MMP)-1 between control subjects and participants with low back pain (LBP). Levels in participants with LBP were analyzed by pooling the epidural steroid injection and surgery cohorts. Analysis of serum levels of (**c**) IL-6 and (**d**) MMP-1 in participants with LBP based on disease severity, classified by Pfirmann grade, where grade 2 represents low disease severity and grade 5 represents high disease severity. Each symbol represents values from a single subject; midline represents the mean; and error bars represent the standard error. *p < 0.05, **p < 0.005

Levels of IL-6, IL-8, IL-10, TNF-α, IFN-γ, MMP-1, MMP-3, and MMP-9 were detectable in nearly all samples (>98 % detected) (Table 1). Controlling for age and gender effects, participants with LBP (combined ESI and surgery cohorts) had serum IL-6 levels (0.52 ± 0.041 pg/ml) that were 41 % higher than those of control participants (0.37 ± 0.057 pg/ml; *p* = 0.049) (Fig. 1a). Serum levels of MMP-1 (14,923 ± 1088 pg/ml) were 56 % lower in LBP participants than in controls (26,264 ± 3828 pg/ml; *p* = 0.0008) (Table 4, Fig. 1b). DDD severity in LBP participants was determined using the Pfirrmann grade (Grades 2–5) of each participant’s MRI scans. Serum levels of IL-6 and MMP-1 were similar across the severity grades (Fig. 1c, d), with no significant differences found in these biochemical factors on the basis of imaging analysis of disease severity grade. Levels of IL-1β, IL-2, IL-4, IL-12p70, and IL-13 were detectable in a small percentage of samples (9–59 % detected) (Table 1). Levels of IL-2 were significantly lower in LBP subjects than in controls, and levels of IL-4 were statistically higher in the LBP cohort than in controls (Table 4).

**Table 4:** Serum biochemical factor levels of control or LBP subjects (geometric mean ± corresponding standard error, pg/ml)

Biochemical factor	Control levels (pg/ml)	LBP levels (ESI & Surgery, pg/ml)	p-value
IL-1β	0.028 ± 0.004	0.033 ± 0.003	0.34
IL-2	0.14 ± 0.026	0.068 ± 0.006	0.0005***
IL-4	0.012 ± 0.002	0.018 ± 0.001	0.012*
IL-6	0.37 ± 0.057	0.52 ± 0.041	0.049*
IL-8	12.49 ± 2.34	10.11 ± 0.95	0.32
IL-10	0.25 ± 0.040	0.23 ± 0.018	0.71
IL-12p70	0.075 ± 0.012	0.089 ± 0.007	0.39
IL-13	0.29 ± 0.039	0.34 ± 0.022	0.39
TNF-α	2.05 ± 0.21	2.47 ± 0.13	0.11
IFN-γ	5.06 ± 0.97	4.86 ± 0.47	0.86
MMP-1	26,264 ± 3,828	14,923 ± 1,088	0.0008**
MMP-3	14,612 ± 1,862	15,440 ± 984	0.70
MMP-9	169,618± 21,665	151,437 ± 9,679	0.43

Statistical analysis (p-value) of differences in levels between control and LBP participants was performed using ANCOVA controlling for age and gender effects

*p<0.05, **p<0.005, ***p<0.0005

**Fig. 2:**
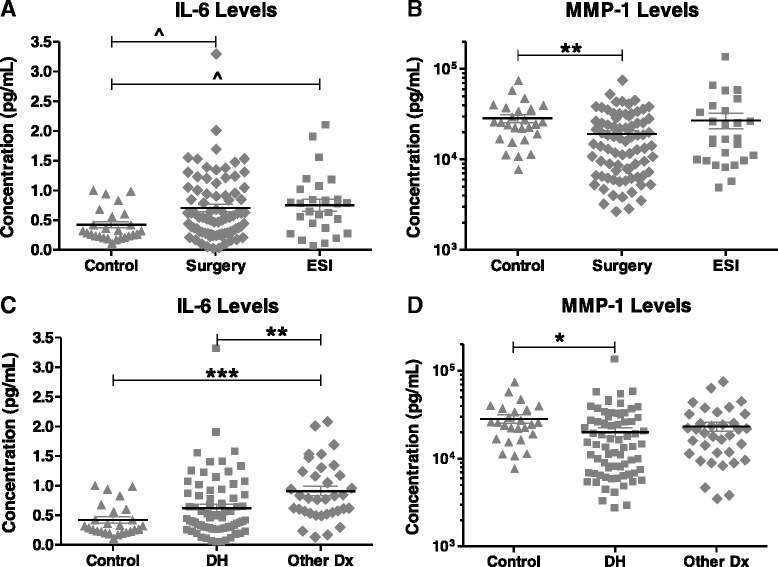
Comparison of serum levels of (**a**) interleukin (IL)-6 and (**b**) matrix metalloproteinase (MMP)-1 between control, surgery, and epidural steroid injection (ESI) cohorts. Comparison of serum levels of (**c**) IL-6 and (**d**) MMP-1 in participants with low back pain by diagnosis. *DH* disc herniation, *Other Dx* other diagnoses including spinal stenosis and degenerative disc disease. Each symbol represents values from a single subject; midline represents the mean; and error bars represent the standard error. *p < 0.05, **p < 0.005, ***p < 0.0005, ^*p* = 0.08

We also compared biochemical levels between control participants and participants in each treatment cohort (surgery or ESI). IL-6 had a trend for higher levels in the surgery and ESI cohorts than in controls (*p* = 0.08), with no significant difference between the two treatment groups (Fig. 2a). MMP-1 levels were 54 % lower in the surgery cohort than in controls (*p* < 0.003), with no significant difference between the two treatment groups (Fig. 2b). All other analytes measured in the multiplex assays were found to be comparable between treatment cohorts.

### Relationships between diagnoses and biochemical levels

Because differences in diagnoses were observed between treatment cohorts, an analysis was performed to compare the levels of serum biochemical factors based on diagnoses. A comparative analysis of the demographics and comorbidities between diagnostic groups is presented in Table 5. No significant differences were found in gender distribution or BMI between DH and Other Dx (Table 5). However, significant differences were observed in subject age and duration of symptoms between diagnostic groups. Participants with LBP due to Other Dx had a higher mean age and longer mean duration of symptoms (36 months) than DH participants (16 months; *p* < 0.0002) (Table 5). Controlling for effects of age, gender, and OA history, participants with SS or DDD (Other Dx) were found to have significantly higher levels of IL-6 than participants with DH (Table 6). Mean serum IL-6 levels in Other Dx (0.74 ± 0.116 pg/ml) were 57 % higher than levels measured in DH subjects (0.47 ± 0.060 pg/ml; *p* < 0.02) (Fig. 2c). MMP-1 levels were significantly lower in DH subjects than in controls, though no significant differences were observed between DH and Other Dx participants (Table 6, Fig. 2d). Changes in levels of IL-13 by diagnosis varied based on gender. In males, IL-13 levels were significantly higher in DH than in Other Dx (*p* < 0.05), though no significant difference was observed in female subjects by diagnosis (Table 6). No other biochemical factor was found to have significant interaction between gender and diagnosis group.

**Table 5:** Demographics factors in LBP participants based on diagnostic group

	DH	Other Dx	p-value
Number of subjects	72	35	
Age (mean ± SD)	47 ± 14	58 ± 13	0.001**
Gender (% male, % female)	39%, 61%	37%, 63%	1.00
BMI (mean ± SD)	28.8 ± 6.0	29.5 ± 5.2	0.32
Duration of Symptoms (months, mean ± SD )	16 ± 31	36 ± 46	0.0002***
Smoking History (pk yrs, mean ± SD)	5 ± 9.2	10.5 ± 16.4	0.13
OA History (number of subjects, % of cohort)	9 (13%)	9 (26%)	0.10

*DH*: Disc Herniation; *Other Dx*: Other diagnoses including spinal stenosis (SS) and degenerative disc disease (DDD). Statistical analysis of differences between DH and Other Dx are presented with p-value

**p<0.005, ***p<0.0005

**Table 6:** Geometric Mean ± corresponding standard error (pg/ml) of serum biochemical factors are reported and compared based on diagnostic groups

		DH	Other Dx	p-value
Cytokines (pg/mL)				
IL-1β		0.033 ± 0.004	0.037 ± 0.005	0.52
IL-2		0.067 ± 0.011	0.064 ± 0.013	0.83
IL-4		0.017± 0.002	0.019 ± 0.003	0.30
IL-6		0.47 ± 0.060	0.74 ± 0.116	0.014*
IL-8		9.57 ± 1.48	12.68 ± 2.43	0.20
IL-10		0.22 ± 0.030	0.27 ± 0.046	0.25
IL-12p70		0.11 ± 0.015	0.079 ± 0.014	0.14
IL-13	Male	0.43 ± 0.059	0.28 ± 0.050	0.046*
	Female	0.32 ± 0.037	0.44 ± 0.067	0.081
TNF-α		2.33 ± 0.20	2.80 ± 0.30	0.14
IFN-γ		4.74 ± 0.73	6.16 ± 1.19	0.23
MMPs (pg/ml)				
MMP-1		15,918 ± 1911	17,730 ± 2650	0.53
MMP-3		16,471 ± 1799	16,304 ± 2219	0.95
MMP-9		154,271 ± 15,925	145,514 ± 18,705	0.69

*DH*: disc herniation; *Other Dx*: other diagnoses include spinal stenosis (SS) and degenerative disc disease (DDD). Statistical analysis (p-value) of differences between DH and Other Dx was performed with ANCOVA, controlling for effects of age, gender and OA history

*p<0.05.

### Correlations between IL-6 and MMP-1 levels and clinical metrics

**Fig. 3:**
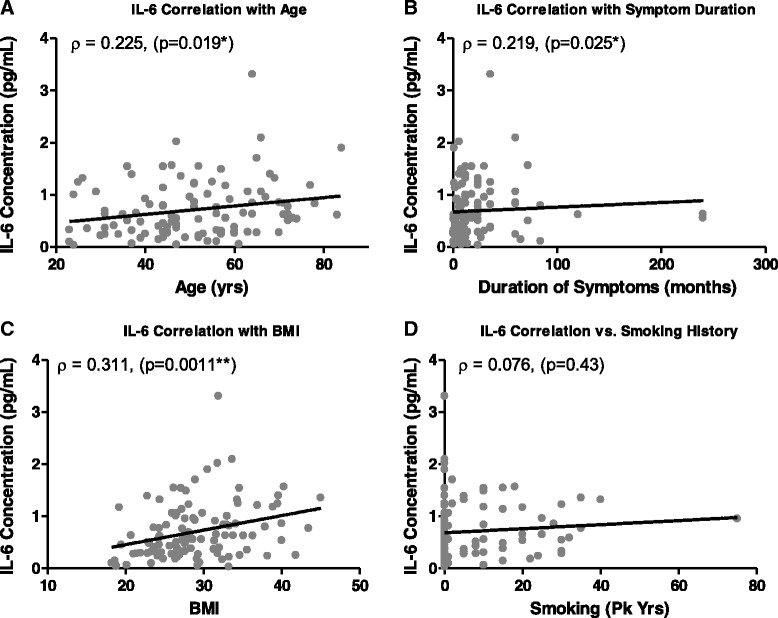
Spearman’s correlation coefficients between interleukin (IL)-6 levels and (**a**) age, (**b**) symptom duration, (**c**) body mass index (BMI), or (**d**) smoking history. The resulting Spearman’s correlation coefficient (ρ) and corresponding *p* value are reported

**Fig. 4:**
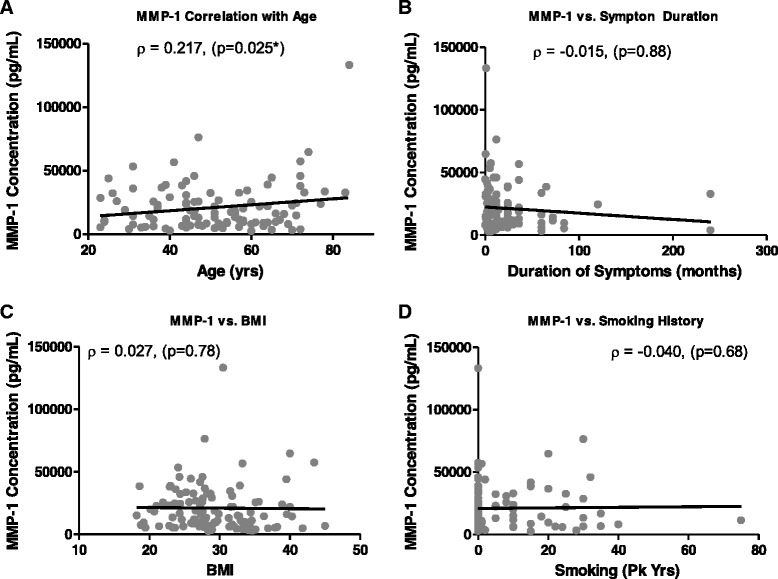
Spearman’s correlation coefficients between matrix metalloproteinase (MMP)-1 levels and (**a**) age, (**b**) symptom duration, (**c**) body mass index (BMI), or (**d**) smoking history. The resulting Spearman’s correlation coefficient (ρ) and corresponding *p* value are reported

Spearman’s correlation coefficients were used to determine the relationship between IL-6 or MMP-1 levels with age, BMI, symptom duration, or smoking history. IL-6 levels in subjects with LBP were found to be significantly correlated with BMI (ρ = 0.311; *p* < 0.005), symptom duration (ρ = 0.219; *p* < 0.05), and age (ρ = 0.225; *p* < 0.05) (Fig. 3). MMP-1 levels were found to be significantly correlated with subject age (ρ = 0.217; *p* < 0.05) (Fig. 4). No significant correlation was observed between MMP-1 and BMI or symptom duration (Fig. 4). Smoking history did not significantly correlate with either IL-6 or MMP-1 levels (Figs. 3d, 4d).

## Discussion

The goals of this study were to examine circulating levels of inflammatory and degenerative mediators associated with IVD disease and to evaluate changes in serum levels based on clinical factors, including diagnosis, age, gender, duration of symptoms, and two features associated with poor outcomes of treatment of LBP- BMI and tobacco use. Our findings indicate that the serum level of IL-6 is significantly higher in participants with LBP. Participants with LBP due to SS or DDD (Other Dx) had significantly higher levels of IL-6 than participants with DH and controls. Positive correlations were found between IL-6 levels and BMI, symptom duration, and age. We also observed that MMP-1 levels were lower in subjects with LBP, specifically subjects with DH, relative to controls. MMP-1 levels were found to be positively correlated with age. We did not observe significant differences in IL-6 or MMP-1 serum levels with respect to disease severity as classified by Pfirrmann grade. MRI findings have been shown to have poor correlation with symptomatic degeneration and do not correlate to functional impairment or pain [56, 57].

To evaluate the significance of cytokine modulation in pathological conditions, it is necessary to establish the physiological range of these molecules in healthy subjects. Serum cytokine levels vary by age, gender, sample type, and type of assay used, among many other variables. The levels of IL-6 measured in the present study are within the reported range for normal human sera, suggesting that LBP subjects have low-grade systemic inflammation. Elevation of circulating IL-6 levels in participants with DDD (part of Other Dx) compared with DH are consistent with observed differences in disc tissue cytokine levels based on diagnosis [58]. Differential protein levels of IL-6 in tissues from participants with DH or DDD have been observed in vitro, with higher levels in samples from subjects with confirmed discogenic pain caused by DDD than in those with DH or sciatica [58]. While we did not segregate patients based on the nature of pain (i.e., discogenic, neuropathic, or mixed), the findings based on diagnosis suggest that patients with discogenic back pain due to DDD or SS may have higher circulating levels of IL-6 than patients with radicular or neuropathic pain associated with herniated discs. Nevertheless, the direct relationship between IVD tissue and blood levels of IL-6 is unknown and future studies investigating the relationship between local (i.e. disc) and circulating cytokine levels are warranted to more conclusively identify the source of elevated systemic cytokine levels. An elevated circulating level of IL-6 represents the complex role of this cytokine in disc diseases and pain. IL-6 can be spontaneously produced in vitro by human herniated and degenerated (i.e., nonherniated) discs [36, 44], and genetic variations in IL-6 are associated with increased risk for IVD disease and back pain [59–61]. IL-6 stimulation of human NP cells downregulates ECM gene expression and PG synthesis [62]. In preclinical models, IL-6 increases production of TNF-α by neurons in dorsal root ganglia and sensitizes dorsal root ganglia to painful stimuli [63, 64]. IL-6 expression levels increase in response to IVD injury in preclinical models [65, 66], and inhibition of IL-6 signaling may be a potential target for analgesic treatment of DDD [65].

In clinical studies, higher serum levels of IL-6 in participants with DH with reported higher pain scores were found relative to participants with lower pain scores [48]. The findings of the present clinical study are the first to be based on examination of circulating cytokine levels in DDD and SS and provide evidence for a more extensive role of IL-6 in disc disease, where patients with DDD or SS have even higher serum cytokine levels than those with DH or control participants. While subjects with a current or past history of OA were not excluded from enrollment in this study, our findings indicate that OA history does not have an appreciable effect on the findings of significantly elevated levels of IL-6 in LBP subjects or on the relative differences observed based on diagnosis of LBP. It is unknown if circulating cytokines are causative of degenerative changes and pain or whether elevated cytokine levels are a consequence of the degeneration and painful condition. Elevated serum IL-6 levels at presentation have also been associated with worse outcomes in recovery from DH at 1-year follow-up [49]. While the effects of elevated serum cytokines in SS or DDD on clinical outcomes are unknown, we speculate that elevated IL-6 levels may be similarly associated with poorer outcomes. For example, low-grade systemic inflammation may promote positive feedback in proinflammatory signaling and degradative enzymes, leading to matrix impairment and bone remodeling, potentially creating an environment that exacerbates degenerative changes.

There are multiple sources of IL-6 secretion and activation in disc-related diseases. In DH, an inflammatory immune response is expected following the protrusion of an immune-privileged NP tissue. In a pilot study consistent with this theory, Kraychete et al. found elevated levels of serum IL-6 in participants with DH compared with controls [67]. In the present study, IL-6 levels in the DH group were on average higher than control levels, though this finding did not achieve statistical significance in a comparison of three groups (DH, Other Dx, and control). The lack of a significant difference in IL-6 levels between the control and DH cohorts may be due to the contributions of covariates, such as age, gender, and OA history, which were controlled for in the present study but not in the study by Kraychete et al. [67]. Cells from degenerated (i.e., nonherniated) discs secrete increased levels of proinflammatory cytokines [44]. Stress concentrations and changes in nondisc tissues of the lumbar spine contribute to the degenerative etiology. In SS, hypertrophy and calcification of spinal ligaments may compromise the adjacent spinal canal and neural foramina. Vertebral bones enlarge due to increased bone stress and cause bone spurs, which can encroach on the spinal canal. Hypertrophy of spinal ligaments, overgrowth of bone, and formation of bone spurs may all contribute to the increased systemic levels of IL-6 in SS and DDD (Other Dx). While weakness in outer IVD is a prerequisite for DH to occur, it is not typically associated with bone spur formation or ligament hypertrophy. Interestingly, some studies have reported elevated circulating levels of TNF-α in subjects with LBP relative to control subjects [67, 68]. Our findings suggest that while raw TNF-α levels may be elevated, modeling contributions of age, gender, and OA history attenuates potential differences in TNF-α levels between LBP and control subjects.

We observed lower levels of MMP-1 in subjects with LBP, specifically due to DH, than in control subjects. In disc tissue, expression of MMP-1 is enhanced in degenerated tissue compared with healthy human disc tissue, and MMP-1 expression by disc cells increases with increasing disease severity [17–20, 69]. The lower serum levels of MMP-1 observed in this study may be indicative of misregulation of MMP-1 levels or activity, confounded by activity of the natural inhibitors of MMP-1 (e.g., tissue inhibitor of metalloproteinases [TIMPs]) or the numerous substrates on which MMP-1 acts [70, 71]. Misregulated levels of many MMPs are observed in many diseases that are characterized by or associated with inflammation. For example, serum MMP-1 levels have been found to be elevated in individuals with disease involving tissue turnover and remodeling, such as OA and RA, compared with healthy controls [72]. Serum MMP-3 levels did not differ between OA patients and normal sera, however, but were elevated in sera from patients with RA [72]. While decreased MMP-1 levels may imply reduced degradation compared with subjects with higher levels, net collagenolytic activity is determined more by the balance between TIMPs and MMPs. Some studies suggest that systemic levels of MMPs vary in individuals on the basis of MMP polymorphisms, where polymorphisms in the MMP-1 gene confer a functional effect on circulating levels of MMP-1 in RA and other diseases [73–76]. Polymorphisms of MMP-1 have been associated with an increased risk of developing lumbar disc disease and may contribute to LBP, sciatica, and disability after lumbar DH [77–79]. Further studies investigating the relationship between local (i.e., disc) and systemic MMP and cytokine levels are warranted to more conclusively identify the function of decreased MMP-1 levels and source of elevated IL-6 levels.

Biomarkers are objective, measurable tools that can predict the incidence or staging of disease, and they have been validated in the context of several diseases. Pain is an intensely subjective experience, and depression is a frequent comorbidity of persistent LBP that is more likely to affect subjective pain measures than serum biochemical levels [80]. Participants in this study were recruited from multiple clinical sites and were evaluated for LBP by their treating physicians on the basis of standard clinical practices. Though validated pain questionnaires were not used in subject recruitment, we observed a significant correlation between IL-6 levels and symptom duration, suggesting that IL-6 levels may be associated with pain measurements (e.g., intensity) in study participants. In many investigations of painful conditions, such as fibromyalgia and OA, investigators are trying to identify systemic factors that can be used to aid diagnosis, stage severity, and indicate prognosis. In chronic pain, circulating IL-6 levels have been correlated with pain levels [81]. In individuals with isolated back pain without radiculopathy, Sowa et al. demonstrated associations between serum levels of neuropeptide Y or chondroitin sulfate 846 with pain and pain-related function [82]. The relationship between inflammatory mediators and pain is not exclusive to LBP, as cytokines and chemokines play a critical role in neuropathic pain [83, 84]. Not surprisingly, patients with other diseases associated with neuropathic pain have systemic elevations of cytokines as well. Patients with knee OA were found to have significantly higher serum levels of IL-6 and IL-10 than controls, though levels of TNF-α and IL-8 were not found to be different in knee OA vs. control subjects [85]. Patients with RA also have elevated systemic IL-6 compared with other patients with joint pain [86]. Elevations in serum levels of IL-6 have also been described in association with aging. Numerous studies of healthy older adults show that levels of several cytokines, especially IL-6 and TNF-α, increase with age in the absence of disease or acute infection. Though correlated with age, the etiology of elevated inflammatory markers remains incompletely defined; however, it is attributed to factors such as visceral adiposity, lower sex steroid hormones, smoking, depression, and periodontal diseases associated with aging [87]. IL-6 levels also increase with BMI and obesity in the absence of other disease [88]. The correlations we observed with increasing BMI underscore the role of obesity as a risk factor for LBP [89] and may implicate systemic inflammation in chronic painful conditions. Increased BMI is associated with disc space narrowing and is a positive risk factor for the development of disc degeneration [90, 91]. This may be due to moderate changes in spinal loads with increasing body weight [92], acting in concert with in an inflammatory milieu caused by higher amounts of adipose tissue.

While many physiological or pathological process may alter circulating biochemical levels (e.g., increases in IL-6), biochemical factors with established roles in disc diseases and pain may prove useful, possibly in combination with emerging imaging technology such as T_1ρ_ MRI or with joint metabolism biomarkers measured in urine or serum [93], to define a more specific profile and mechanism of disease. T_1ρ_ MRI provides a measure of functional disc integrity [94–98], shows promise for more sensitive and early identification of disc pathology, and may have stronger correlations with clinical status than standard MRI. Combining cytokine profiles with joint metabolism biomarkers or advanced spinal imaging technology may be useful in developing biomarker profiles of participants with disc diseases of varying etiologies and severities. Discography for the diagnosis of the pain generator level in LBP is now generally in decline due to lack of diagnostic accuracy and sensitivity and because of its invasive nature. Serum cytokine profiling may represent a less invasive approach to evaluation of subjects in the absence of specific disc pathology on clinical imaging. Measures of serum biochemical factors in patients with disc diseases may assist in refining diagnoses and possibly by defining more uniform cohorts of patients with reference to circulating inflammatory levels.

ESI is commonly used for treatment of LBP, where nonspecific immunosuppression may affect inflammatory etiologies of pain. There has been increased use of epidurally injected medications with increased specificity, including U.S. Food and Drug Administration–approved agents that target TNF-α (e.g., infliximab, adalimumab, and etanercept) or IL-6 (tocilizumab). The efficacy of these drugs in treating LBP is still being evaluated, but studies have shown symptomatic improvement for patients with radiculopathy [99], perhaps due to mitigating the effects of inflammation on the dorsal root ganglia [100]. Ohtori et al. reported reduction of radicular leg pain, numbness, and LBP with tocilizumab in patients with SS [101]. However, there are few other studies in which researchers have presented clinical data on LBP due to other diagnoses or in the absence of radiculopathy. The possibility of using serum cytokine measures of IL-6 to predict response to treatment with progressively targeted therapies fits the emerging concept of personalized molecular medicine, where treatments are tailored to an individual’s specific biology through increasingly sensitive detection of molecular abnormalities, and the use of these inflammatory mediators as biomarkers for LBP.

### Study limitations

While statistical significance was observed for IL-2 and IL-4 levels between the control and LBP cohorts, these findings are limited by the fact that only 59 % and 28 % of all samples, respectively, had detectable levels. Similarly, IL-13 levels significantly differed in male subjects by diagnoses; however, IL-13 levels were detectable in only 41 % of samples. The relatively low percentages of detected levels of IL-2, IL-4, and IL-13 are consistent with data provided by the assay manufacturer for measurements in normal human serum. Therefore, caution is warranted in interpreting the findings of significant differences in these factors.

This study included inhomogeneous distributions of participants across treatment or diagnosis cohorts. The number of participants undergoing surgery was greater than those undergoing ESI. Similarly, the diagnoses are not equally represented, with significantly more participants having DH compared with Other Dx. Participants undergoing surgery for DDD are particularly underrepresented (*n* = 2), in part because the indications for surgery with discogenic back pain remain controversial and participants with a history of discogram were excluded. Additionally, comorbidities may potentially affect inflammatory mediators observed in the systemic circulation of control participants.

## Conclusions

Serum levels of IL-6 were significantly higher and levels of MMP-1 were significantly lower in participants with LBP than in control subjects. Participants with LBP due to SS or DDD (Other Dx) had significantly higher levels of IL-6 than participants with DH and control subjects, after controlling for effects of age, gender, and OA history. This finding suggests that circulating proinflammatory cytokines play a more extensive role in disc diseases such as SS and DDD. Positive correlations were found between IL-6 levels and BMI, symptom duration, and age in participants with LBP. MMP-1 levels were also positively correlated with subject age in the LBP cohort. The results of the present study, where circulating levels of IL-6 in participants with DDD (part of Other Dx) were significantly higher than DH, are consistent with observed differences in disc tissue cytokine levels. The observed lower levels of circulating MMP-1 in DH are somewhat unexpected and may represent dysregulation of MMP-1 activity in this diagnosis group. It is unknown if circulating cytokines are causative of degenerative changes and pain or whether elevated cytokine levels are a consequence of the degeneration and pain condition. In future studies, we will evaluate the relationship between cytokine levels and outcomes to treatment of SS and DDD, as described in our recent exploratory study [102].

## Abbreviations

ADAMTS: a disintegrin and metalloproteinase with thrombospondin motifs
AF: annulus fibrosis
ANCOVA: analysis of covariance
BMI: body mass index
DDD: degenerative disc disease
DH: disc herniation
ECM: extracellular matrix
ESI: epidural steroid injection
HSD: honest significant difference
IFN-γ: interferon-γ
IL: interleukin
IVD: intervertebral disc
LBP: low back pain
LLOD: lower limit of detection
MMP: matrix metalloproteinase
MRI: magnetic resonance imaging
NP: nucleus pulposus
OA: osteoarthritis
Other Dx: other diagnoses (DDD and SS)
PG: proteoglycan
RA: rheumatoid arthritis
SS: spinal stenosis
TIMP: tissue inhibitor of metalloproteinase
TNF-α: tumor necrosis factor-α
ULOD: upper limit of detection

## References

[1] Dagenais S, Caro J, Haldeman S (2008). A systematic review of low back pain cost of illness studies in the United States and internationally. Spine J.

[2] United States Bone and Joint Initiative; Bone and Joint Decade (2011). Health care utilization and economic cost of musculoskeletal disease. The burden of musculoskeletal diseases in the United States: prevalence, societal and economic cost.

[3] Ivanova JI, Birnbaum HG, Schiller M, Kantor E, Johnstone BM, Swindle RW (2011). Real-world practice patterns, health-care utilization, and costs in patients with low back pain: the long road to guideline-concordant care. Spine J.

[4] Gore M, Sadosky A, Stacey BR, Tai KS, Leslie D (2012). The burden of chronic low back pain: clinical comorbidities, treatment patterns, and health care costs in usual care settings. Spine (Phila Pa 1976).

[5] Manchikanti L, Singh V, Falco FJ, Benyamin RM, Hirsch JA (2014). Epidemiology of low back pain in adults. Neuromodulation..

[6] Fardon DF, Williams AL, Dohring EJ, Murtagh FR, Gabriel Rothman SL, Sze GK (2014). Lumbar disc nomenclature: version 2.0: recommendations of the combined task forces of the North American Spine Society, the American Society of Spine Radiology, and the American Society of Neuroradiology. Spine (Phila Pa 1976).

[7] Meyer F, Börm W, Thomé C (2008). Degenerative cervical spinal stenosis: current strategies in diagnosis and treatment. Dtsch Arztebl Int.

[8] Siebert E, Pruss H, Klingebiel R, Failli V, Einhaupl KM, Schwab JM (2009). Lumbar spinal stenosis: syndrome, diagnostics and treatment. Nat Rev Neurol.

[9] Macki M, Hernandez-Hermann M, Bydon M, Gokaslan A, McGovern K, Bydon A (2014). Spontaneous regression of sequestrated lumbar disc herniations: literature review. Clin Neurol Neurosurg..

[10] Sekiguchi M, Yonemoto K, Kakuma T, Nikaido T, Watanabe K, Kato K (2015). Relationship between lumbar spinal stenosis and psychosocial factors: a multicenter cross-sectional study (DISTO project). Eur Spine J.

[11] Nerland US, Jakola AS, Giannadakis C, Solheim O, Weber C, Nygaard ØP (2015). The risk of getting worse: predictors of deterioration after decompressive surgery for lumbar spinal stenosis: a multicenter observational study. World Neurosurg.

[12] Antoniou J, Steffen T, Nelson F, Winterbottom N, Hollander AP, Poole RA (1996). The human lumbar intervertebral disc: evidence for changes in the biosynthesis and denaturation of the extracellular matrix with growth, maturation, ageing, and degeneration. J Clin Invest.

[13] Pearce RH, Grimmer BJ, Adams ME (1987). Degeneration and the chemical composition of the human lumbar intervertebral disc. J Orthop Res.

[14] Roughley PJ, Alini M, Antoniou J (2002). The role of proteoglycans in aging, degeneration and repair of the intervertebral disc. Biochem Soc Trans.

[15] Millward-Sadler SJ, Costello PW, Freemont AJ, Hoyland JA (2009). Regulation of catabolic gene expression in normal and degenerate human intervertebral disc cells: implications for the pathogenesis of intervertebral disc degeneration. Arthritis Res Ther.

[16] Keshari KR, Lotz JC, Link TM, Hu S, Majumdar S, Kurhanewicz J (2008). Lactic acid and proteoglycans as metabolic markers for discogenic back pain. Spine (Phila Pa 1976).

[17] Le Maitre CL, Freemont AJ, Hoyland JA (2004). Localization of degradative enzymes and their inhibitors in the degenerate human intervertebral disc. J Pathol.

[18] Le Maitre CL, Freemont AJ, Hoyland JA (2006). Human disc degeneration is associated with increased MMP 7 expression. Biotech Histochem.

[19] Le Maitre CL, Pockert A, Buttle DJ, Freemont AJ, Hoyland JA (2007). Matrix synthesis and degradation in human intervertebral disc degeneration. Biochem Soc Trans.

[20] Weiler C, Nerlich AG, Zipperer J, Bachmeier BE, Boos N (2002). 2002 SSE Award Competition in Basic Science: expression of major matrix metalloproteinases is associated with intervertebral disc degradation and resorption. Eur Spine J.

[21] Wang J, Markova D, Anderson DG, Zheng Z, Shapiro IM, Risbud MV (2011). TNF-α and IL-1β promote a disintegrin-like and metalloprotease with thrombospondin type I motif-5-mediated aggrecan degradation through syndecan-4 in intervertebral disc. J Biol Chem.

[22] Pockert AJ, Richardson SM, Le Maitre CL, Lyon M, Deakin JA, Buttle DJ (2009). Modified expression of the ADAMTS enzymes and tissue inhibitor of metalloproteinases 3 during human intervertebral disc degeneration. Arthritis Rheum.

[23] Tiaden AN, Klawitter M, Lux V, Mirsaidi A, Bahrenberg G, Glanz S (2012). Detrimental role for human high temperature requirement serine protease A1 (HTRA1) in the pathogenesis of intervertebral disc (IVD) degeneration. J Biol Chem.

[24] Akhatib B, Onnerfjord P, Gawri R, Ouellet J, Jarzem P, Heinegård D (2013). Chondroadherin fragmentation mediated by the protease HTRA1 distinguishes human intervertebral disc degeneration from normal aging. J Biol Chem.

[25] Ariga K, Yonenobu K, Nakase T, Kaneko M, Okuda S, Uchiyama Y (2001). Localization of cathepsins D, K, and L in degenerated human intervertebral discs. Spine (Phila Pa 1976).

[26] Konttinen YT, Kääpä E, Hukkanen M, Gu XH, Takagi M, Santavirta S (1999). Cathepsin G in degenerating and healthy discal tissue. Clin Exp Rheumatol.

[27] Sztrolovics R, Alini M, Roughley PJ, Mort JS (1997). Aggrecan degradation in human intervertebral disc and articular cartilage. Biochem J.

[28] Risbud MV, Shapiro IM (2014). Role of cytokines in intervertebral disc degeneration: pain and disc content. Nat Rev Rheumatol.

[29] Roberts S, Evans H, Trivedi J, Menage J (2006). Histology and pathology of the human intervertebral disc. J Bone Joint Surg Am..

[30] Kanayama M, Togawa D, Takahashi C, Terai T, Hashimoto T (2009). Cross-sectional magnetic resonance imaging study of lumbar disc degeneration in 200 healthy individuals. J Neurosurg Spine.

[31] Cheung KM, Karppinen J, Chan D, Ho DW, Song YQ, Sham P (2009). Prevalence and pattern of lumbar magnetic resonance imaging changes in a population study of one thousand forty-three individuals. Spine (Phila Pa 1976).

[32] Battié MC, Videman T, Kaprio J, Gibbons LE, Gill K, Manninen H (2009). The Twin Spine Study: contributions to a changing view of disc degeneration. Spine J.

[33] Adams MA, Freeman BJ, Morrison HP, Nelson IW, Dolan P (2000). Mechanical initiation of intervertebral disc degeneration. Spine (Phila Pa 1976).

[34] Wang D, Nasto LA, Roughley P, Leme AS, Houghton AM, Usas A (2012). Spine degeneration in a murine model of chronic human tobacco smokers. Osteoarthritis Cartilage.

[35] Ganko R, Rao PJ, Phan K, Mobbs RJ (2015). Can bacterial infection by low virulent organisms be a plausible cause for symptomatic disc degeneration? A systematic review. Spine (Phila Pa 1976).

[36] Kang JD, Georgescu HI, McIntyre-Larkin L, Stefanovic-Racic M, Donaldson WF, Evans CH (1996). Herniated lumbar intervertebral discs spontaneously produce matrix metalloproteinases, nitric oxide, interleukin-6, and prostaglandin E2. Spine (Phila Pa 1976).

[37] Kang JD, Stefanovic-Racic M, McIntyre LA, Georgescu HI, Evans CH (1997). Toward a biochemical understanding of human intervertebral disc degeneration and herniation: contributions of nitric oxide, interleukins, prostaglandin E2, and matrix metalloproteinases. Spine (Phila Pa 1976).

[38] Ahn SH, Cho YW, Ahn MW, Jang SH, Sohn YK, Kim HS (2002). mRNA expression of cytokines and chemokines in herniated lumbar intervertebral discs. Spine (Phila Pa 1976).

[39] Le Maitre CL, Hoyland JA, Freemont AJ (2007). Catabolic cytokine expression in degenerate and herniated human intervertebral discs: IL-1β and TNFα expression profile. Arthritis Res Ther.

[40] Bachmeier BE, Nerlich AG, Weiler C, Paesold G, Jochum M, Boos N (2007). Analysis of tissue distribution of TNF-α, TNF-α-receptors, and the activating TNF-α-converting enzyme suggests activation of the TNF-α system in the aging intervertebral disc. Ann N Y Acad Sci..

[41] Shamji MF, Setton LA, Jarvis W, So S, Chen J, Jing L (2010). Proinflammatory cytokine expression profile in degenerated and herniated human intervertebral disc tissues. Arthritis Rheum.

[42] Le Maitre CL, Freemont AJ, Hoyland JA (2005). The role of interleukin-1 in the pathogenesis of human intervertebral disc degeneration. Arthritis Res Ther.

[43] Rand N, Reichert F, Floman Y, Rotshenker S (1997). Murine nucleus pulposus-derived cells secrete interleukins-1-β, -6, and -10 and granulocyte-macrophage colony-stimulating factor in cell culture. Spine (Phila Pa 1976).

[44] Krock E, Rosenzweig DH, Chabot-Dore AJ, Jarzem P, Weber MH, Ouellet JA (2014). Painful, degenerating intervertebral discs up-regulate neurite sprouting and CGRP through nociceptive factors. J Cell Mol Med.

[45] Freemont AJ, Watkins A, Le Maitre C, Baird P, Jeziorska M, Knight MT (2002). Nerve growth factor expression and innervation of the painful intervertebral disc. J Pathol.

[46] Melrose J, Roberts S, Smith S, Menage J, Ghosh P (2002). Increased nerve and blood vessel ingrowth associated with proteoglycan depletion in an ovine anular lesion model of experimental disc degeneration. Spine (Phila Pa 1976).

[47] Brisby H, Olmarker K, Larsson K, Nutu M, Rydevik B (2002). Proinflammatory cytokines in cerebrospinal fluid and serum in patients with disc herniation and sciatica. Eur Spine J.

[48] Pedersen LM, Schistad E, Jacobsen LM, Roe C, Gjerstad J (2015). Serum levels of the pro-inflammatory interleukins 6 (IL-6) and -8 (IL-8) in patients with lumbar radicular pain due to disc herniation: a 12-month prospective study. Brain Behav Immun..

[49] Schistad EI, Espeland A, Pedersen LM, Sandvik L, Gjerstad J, Roe C (2014). Association between baseline IL-6 and 1-year recovery in lumbar radicular pain. Eur J Pain.

[50] Kerr D, Zhao W, Lurie JD (2015). What are long-term predictors of outcomes for lumbar disc herniation? A randomized and observational study. Clin Orthop Relat Res.

[51] Michaud M, Balardy L, Moulis G, Gaudin C, Peyrot C, Vellas B (2013). Proinflammatory cytokines, aging, and age-related diseases. J Am Med Dir Assoc.

[52] Fantuzzi G (2005). Adipose tissue, adipokines, and inflammation. J Allergy Clin Immunol.

[53] Petrescu F, Voican SC, Silosi I (2010). Tumor necrosis factor-α serum levels in healthy smokers and nonsmokers. Int J Chron Obstruct Pulmon Dis..

[54] Pfirrmann CW, Metzdorf A, Zanetti M, Hodler J, Boos N (2001). Magnetic resonance classification of lumbar intervertebral disc degeneration. Spine (Phila Pa 1976).

[55] Stein A, Panjwani A, Sison C, Rosen L, Chugh R, Metz C (2013). Pilot study: elevated circulating levels of the proinflammatory cytokine macrophage migration inhibitory factor in patients with chronic spinal cord injury. Arch Phys Med Rehabil.

[56] Mirza SK, Deyo RA (2007). Systematic review of randomized trials comparing lumbar fusion surgery to nonoperative care for treatment of chronic back pain. Spine (Phila Pa 1976).

[57] DeVine J, Norvell DC, Ecker E, Fourney DR, Vaccaro A, Wang J (2011). Evaluating the correlation and responsiveness of patient-reported pain with function and quality-of-life outcomes after spine surgery. Spine (Phila Pa 1976).

[58] Burke JG, Watson RW, McCormack D, Dowling FE, Walsh MG, Fitzpatrick JM (2002). Intervertebral discs which cause low back pain secrete high levels of proinflammatory mediators. J Bone Joint Surg Br.

[59] Noponen-Hietala N, Virtanen I, Karttunen R, Schwenke S, Jakkula E, Li H (2005). Genetic variations in IL6 associate with intervertebral disc disease characterized by sciatica. Pain.

[60] Karppinen J, Daavittila I, Noponen N, Haapea M, Taimela S, Vanharanta H (2008). Is the interleukin-6 haplotype a prognostic factor for sciatica?. Eur J Pain.

[61] Kelempisioti A, Eskola PJ, Okuloff A, Karjalainen U, Takatalo J, Daavittila I (2011). Genetic susceptibility of intervertebral disc degeneration among young Finnish adults. BMC Med Genet..

[62] Studer RK, Vo N, Sowa G, Ondeck C, Kang J (2011). Human nucleus pulposus cells react to IL-6: independent actions and amplification of response to IL-1 and TNF-α. Spine (Phila Pa 1976).

[63] Murata Y, Rydevik B, Nannmark U, Larsson K, Takahashi K, Kato Y (2011). Local application of interleukin-6 to the dorsal root ganglion induces tumor necrosis factor-α in the dorsal root ganglion and results in apoptosis of the dorsal root ganglion cells. Spine (Phila Pa 1976).

[64] Wei XH, Na XD, Liao GJ, Chen QY, Cui Y, Chen FY (2013). The up-regulation of IL-6 in DRG and spinal dorsal horn contributes to neuropathic pain following L5 ventral root transection. Exp Neurol..

[65] Sainoh T, Orita S, Miyagi M, Sakuma Y, Yamauchi K, Suzuki M (2015). Interleukin-6 and interleukin-6 receptor expression, localization, and involvement in pain-sensing neuron activation in a mouse intervertebral disc injury model. J Orthop Res.

[66] Walter BA, Korecki CL, Purmessur D, Roughley PJ, Michalek AJ, Iatridis JC (2011). Complex loading affects intervertebral disc mechanics and biology. Osteoarthritis Cartilage.

[67] Kraychete DC, Sakata RK, Issy AM, Bacellar O, Santos-Jesus R, Carvalho EM (2010). Serum cytokine levels in patients with chronic low back pain due to herniated disc: analytical cross-sectional study. Sao Paulo Med J.

[68] Wang H, Schiltenwolf M, Buchner M (2008). The role of TNF-α in patients with chronic low back pain – a prospective comparative longitudinal study. Clin J Pain.

[69] Bachmeier BE, Nerlich A, Mittermaier N, Weiler C, Lumenta C, Wuertz K (2009). Matrix metalloproteinase expression levels suggest distinct enzyme roles during lumbar disc herniation and degeneration. Eur Spine J.

[70] Parks WC, Wilson CL, Lopez-Boado YS (2004). Matrix metalloproteinases as modulators of inflammation and innate immunity. Nat Rev Immunol.

[71] Manicone AM, McGuire JK (2008). Matrix metalloproteinases as modulators of inflammation. Semin Cell Dev Biol.

[72] Mahmoud RK, El-Ansary AK, El-Eishi HH, Kamal HM, El-Saeed NH (2005). Matrix metalloproteinases MMP-3 and MMP-1 levels in sera and synovial fluids in patients with rheumatoid arthritis and osteoarthritis. Ital J Biochem.

[73] Chen Y, Nixon NB, Dawes PT, Mattey DL (2012). Influence of variations across the MMP-1 and -3 genes on the serum levels of MMP-1 and -3 and disease activity in rheumatoid arthritis. Genes Immun.

[74] Dey S, Ghosh N, Saha D, Kesh K, Gupta A, Swarnakar S (2014). Matrix metalloproteinase-1 (MMP-1) promoter polymorphisms are well linked with lower stomach tumor formation in eastern Indian population. PLoS One.

[75] Cheng YC, Kao WH, Mitchell BD, O’Connell JR, Shen H, McArdle PF (2009). Genome-wide association scan identifies variants near *Matrix Metalloproteinase* (*MMP*) genes on chromosome 11q21-22 strongly associated with serum MMP-1 levels. Circ Cardiovasc Genet.

[76] Montes AH, Valle-Garay E, Alvarez V, Pevida M, García Pérez E, Paz J (2010). A functional polymorphism in MMP1 could influence osteomyelitis development. J Bone Miner Res.

[77] Song YQ, Ho DW, Karppinen J, Kao PY, Fan BJ, Luk KD (2008). Association between promoter −1607 polymorphism of *MMP1* and lumbar disc disease in Southern Chinese. BMC Med Genet..

[78] Jacobsen LM, Schistad EI, Storesund A, Pedersen LM, Espeland A, Rygh LJ (2013). The MMP1 rs1799750 2G allele is associated with increased low back pain, sciatica, and disability after lumbar disk herniation. Clin J Pain.

[79] Mayer JE, Iatridis JC, Chan D, Qureshi SA, Gottesman O, Hecht AC (2013). Genetic polymorphisms associated with intervertebral disc degeneration. Spine J.

[80] Wang H, Ahrens C, Rief W, Gantz S, Schiltenwolf M, Richter W (2010). Influence of depression symptoms on serum tumor necrosis factor-α of patients with chronic low back pain. Arthritis Res Ther.

[81] Koch A, Zacharowski K, Boehm O, Stevens M, Lipfert P, von Giesen HJ (2007). Nitric oxide and pro-inflammatory cytokines correlate with pain intensity in chronic pain patients. Inflamm Res.

[82] Sowa GA, Perera S, Bechara B, Agarwal V, Boardman J, Huang W (2014). Associations between serum biomarkers and pain and pain-related function in older adults with low back pain: a pilot study. J Am Geriatr Soc.

[83] Clark AK, Old EA, Malcangio M (2013). Neuropathic pain and cytokines: current perspectives. J Pain Res..

[84] Kiguchi N, Kobayashi Y, Kishioka S (2012). Chemokines and cytokines in neuroinflammation leading to neuropathic pain. Curr Opin Pharmacol.

[85] Imamura M, Ezquerro F, Marcon Alfieri F, Vilas Boas L, Tozetto-Mendoza TR, Chen J (2015). Serum levels of proinflammatory cytokines in painful knee osteoarthritis and sensitization. Int J Inflam..

[86] Arvidson NG, Gudbjörnsson B, Elfman L, Rydén AC, Tötterman TH, Hällgren R (1994). Circadian rhythm of serum interleukin-6 in rheumatoid arthritis. Ann Rheum Dis.

[87] Singh T, Newman AB (2011). Inflammatory markers in population studies of aging. Ageing Res Rev.

[88] Olszanecka-Glinianowicz M, Zahorska-Markiewicz B, Janowska J, Zurakowski A (2004). Serum concentrations of nitric oxide, tumor necrosis factor (TNF)-α and TNF soluble receptors in women with overweight and obesity. Metabolism.

[89] Van Nieuwenhuyse A, Crombez G, Burdorf A, Verbeke G, Masschelein R, Moens G (2009). Physical characteristics of the back are not predictive of low back pain in healthy workers: a prospective study. BMC Musculoskelet Disord..

[90] Samartzis D, Karppinen J, Chan D, Luk KD, Cheung KM (2012). The association of lumbar intervertebral disc degeneration on magnetic resonance imaging with body mass index in overweight and obese adults: a population-based study. Arthritis Rheum.

[91] Urquhart DM, Kurniadi I, Triangto K, Wang Y, Wluka AE, O’Sullivan R (2014). Obesity is associated with reduced disc height in the lumbar spine but not at the lumbosacral junction. Spine (Phila Pa 1976).

[92] Hajihosseinali M, Arjmand N, Shirazi-Adl A (2015). Effect of body weight on spinal loads in various activities: a personalized biomechanical modeling approach. J Biomech.

[93] Goode AP, Marshall SW, Kraus VB, Renner JB, Sturmer T, Carey TS (2012). Association between serum and urine biomarkers and lumbar spine individual radiographic features: the Johnston County Osteoarthritis Project. Osteoarthritis Cartilage.

[94] Blumenkrantz G, Zuo J, Li X, Kornak J, Link TM, Majumdar S (2010). In vivo 3.0-Tesla magnetic resonance *T*_1ρ_ and *T*_2_ relaxation mapping in subjects with intervertebral disc degeneration and clinical symptoms. Magn Reson Med.

[95] Filippi CG, Duncan CT, Watts R, Nickerson JP, Gonyea JV, Hipko SG (2013). In vivo quantification of T1ρ in lumbar spine disk spaces at 3 T using parallel transmission MRI. AJR Am J Roentgenol.

[96] Johannessen W, Auerbach JD, Wheaton AJ, Kurji A, Borthakur A, Reddy R (2006). Assessment of human disc degeneration and proteoglycan content using T_1ρ_-weighted magnetic resonance imaging. Spine (Phila Pa 1976).

[97] Nguyen AM, Johannessen W, Yoder JH, Wheaton AJ, Vresilovic EJ, Borthakur A (2008). Noninvasive quantification of human nucleus pulposus pressure with use of T1ρ-weighted magnetic resonance imaging. J Bone Joint Surg Am.

[98] Regatte RR, Akella SV, Borthakur A, Kneeland JB, Reddy R (2002). Proteoglycan depletion-induced changes in transverse relaxation maps of cartilage: comparison of T2 and T1ρ. Acad Radiol.

[99] Pimentel DC, El Abd O, Benyamin RM, Buehler AM, Leite VF, Mazloomdoost D (2014). Anti-tumor necrosis factor antagonists in the treatment of low back pain and radiculopathy: a systematic review and meta-analysis. Pain Physician.

[100] Andrade P, Hoogland G, Del Rosario JS, Steinbusch HW, Visser-Vandewalle V, Daemen MA (2014). Tumor necrosis factor-α inhibitors alleviation of experimentally induced neuropathic pain is associated with modulation of TNF receptor expression. J Neurosci Res.

[101] Ohtori S, Miyagi M, Eguchi Y, Inoue G, Orita S, Ochiai N (2012). Efficacy of epidural administration of anti-interleukin-6 receptor antibody onto spinal nerve for treatment of sciatica. Eur Spine J.

[102] Weber KT, Satoh S, Alipui DO, Virojanapa J, Levine M, Sison C, etal. Exploratory study for identifying systemic biomarkers that correlate with pain response in patients with intervertebral disc disorders. Immunol Res. 2015;63(1-3):170-80. doi: 10.1007/s12026-015-8709-2. PMID: 26440592. 10.1007/s12026-015-8709-2 PMC4689741 26440592

